# The cardiac lymphatic system stimulates resolution of inflammation following myocardial infarction

**DOI:** 10.1172/JCI97192

**Published:** 2018-07-09

**Authors:** Joaquim Miguel Vieira, Sophie Norman, Cristina Villa del Campo, Thomas J. Cahill, Damien N. Barnette, Mala Gunadasa-Rohling, Louise A. Johnson, David R. Greaves, Carolyn A. Carr, David G. Jackson, Paul R. Riley

**Affiliations:** 1 Burdon-Sanderson Cardiac Science Centre, Department of Physiology, Anatomy and Genetics,; 2 MRC Human Immunology Unit, Weatherall Institute of Molecular Medicine, John Radcliffe Hospital,; 3 Sir William Dunn School of Pathology, University of Oxford, Oxford, United Kingdom.

**Keywords:** Inflammation, Vascular Biology, Cardiovascular disease

## Abstract

Myocardial infarction (MI) arising from obstruction of the coronary circulation engenders massive cardiomyocyte loss and replacement by non-contractile scar tissue, leading to pathological remodeling, dysfunction, and ultimately heart failure. This is presently a global health problem for which there is no effective cure. Following MI, the innate immune system directs the phagocytosis of dead cell debris in an effort to stimulate cell repopulation and tissue renewal. In the mammalian adult heart, however, the persistent influx of immune cells, coupled with the lack of an inherent regenerative capacity, results in cardiac fibrosis. Here, we reveal that stimulation of cardiac lymphangiogenesis with VEGF-C improves clearance of the acute inflammatory response after MI by trafficking immune cells to draining mediastinal lymph nodes (MLNs) in a process dependent on lymphatic vessel endothelial hyaluronan receptor 1 (LYVE-1). Deletion of *Lyve1* in mice, preventing docking and transit of leukocytes through the lymphatic endothelium, results in exacerbation of chronic inflammation and long-term deterioration of cardiac function. Our findings support targeting of the lymphatic/immune cell axis as a therapeutic paradigm to promote immune modulation and heart repair.

## Introduction

The lymphatic system is a network of vessels that pervades the entire body, complementary to the blood cardiocirculatory system. It comprises blind-ended capillaries that collect extravasated interstitial fluid, macromolecules, and leukocytes from the periphery of the body, preventing their accumulation in tissues, and transports them via lymph through larger collecting afferent vessels toward draining lymph nodes (1). These are secondary lymphoid organs, enriched for naive lymphocytes, that filter the lymph to detect foreign particles (antigens), triggering lymphocyte activation and an adaptive immune response (1, 2). Lymph then flows from the nodes via efferent lymphatic vessels into larger lymphatic ducts, eventually being returned to the blood circulation, at the level of the subclavian veins. The systemic lymphatic network thus plays a critical role in tissue fluid regulation and immune surveillance during homeostasis (1).

Lymphatic vessel growth, or lymphangiogenesis, begins early during embryogenesis and is promoted by VEGF-C and VEGF-D signaling through their cognate receptor, VEGFR-3, which is expressed in both blood and lymphatic endothelium and becomes specified to the latter toward the end of development (1, 3). Postnatally, lymphatic expansion is associated with wound healing and chronic inflammation (4). Previously, the majority of the studies have focused on peripheral tissue injury (for example, skin wounding; ref. 5), whereby following initiation of the inflammatory process, lymphatic endothelial cells (LECs) proliferate in response to VEGF-C and VEGF-D, leading to network expansion (reviewed in ref. 4). In this setting, activated LECs also secrete chemokines, including CCL21, to attract antigen-charged leukocytes and promote their trafficking via lymph, leading to amplification of the immune response (6). As inflammation resolves, anti-lymphangiogenic factors accumulate (e.g., T lymphocytes [ref. 7] and TGF-β [ref. 8]), and the newly expanded lymphatic vasculature regresses.

The mammalian heart has an extensive lymphatic capillary network that drains lymph to periaortic and paratracheal mediastinal lymph nodes (MLNs) via subepicardial pre-collector vessels (9). This vascular network ensures optimal heart function, as an increase in tissue fluid content by as little as 2.5% can lead to 30%–40% reduction in cardiac output (10, 11). Cardiac lymphangiogenesis has been described during heart development and in response to injury (myocardial infarction [MI]), whereby induced lymphatic sprouting resulted in improved prognosis (12–14) via an uncharacterized mechanism. MI triggers a stereotypical reaction of the innate immune system, involving an initial influx to the injury site of a substantial number of neutrophils attracted by apoptotic signals released by dying cells (e.g., IL-1α; ref. 15), cell debris, and cytokines from neighboring cells (16). This acute response is critical to avoiding the undesirable consequences of accumulating damage signals in situ and to restoring tissue homeostasis. However, neutrophils generate high levels of ROS, secrete matrix-degrading proteases, and undergo apoptosis following phagocytosis. Consequently, neutrophils within the injured milieu require timely suppression and spatial confinement to prevent exacerbation of tissue damage, most notably the death of viable cardiomyocytes and enhanced extracellular matrix degradation, that results in chamber dilatation, remodeling, and fibrotic scarring (16). Circulating monocytes and activated macrophages represent the second wave of innate immune cells. These proinflammatory (so-called M1) monocytes/macrophages participate in clearance of cell debris, including apoptotic neutrophils and cardiomyocytes, by essential efferocytosis (17) and also secrete ROS, proteases, and cytokines, leading to pathological remodeling of the infarcted tissue (16). Subsequently M1 macrophages adopt an antiinflammatory, pro-reparative M2 phenotype and contribute to ECM remodeling and fibrosis by signaling to myofibroblasts to patch the area of injury and prevent wall rupture after MI (16, 18).

Given the physiological role of the lymphatics in immune surveillance and their response to inflammation at sites of skin wounding (5, 6, 19), we sought to investigate the mechanism by which VEGF-C–induced lymphangiogenesis improves functional outcome after MI and, for the first time to our knowledge, translate findings from peripheral wound healing to organ-based repair. Specifically, we demonstrate that the expanded lymphatic network, draining the injury area, enables active trafficking of innate immune cells (neutrophils, macrophages, and dendritic cells) via lymph to regional MLNs, in a process dependent on the lymphatic endothelial receptor lymphatic vessel endothelial hyaluronan (HA) receptor 1 (LYVE-1). Critically, a global reduction in immune cell load within the infarcted milieu is sufficient to favor optimal heart repair, whereas in *Lyve1^–/–^* mutant mice immune cell trafficking and clearance to MLNs are blocked, resulting in a loss of viable myocardium, enhanced scarring, and significantly reduced cardiac output. These findings are of widespread interest in terms of understanding how to optimize immune cell clearance from the site of cardiac injury and to offset the chronic progression to pathological remodeling, functional impairment, and ultimately heart failure.

## Results

### VEGF-C–driven cardiac lymphangiogenesis increases clearance of immune cells after MI.

**Figure 1:**
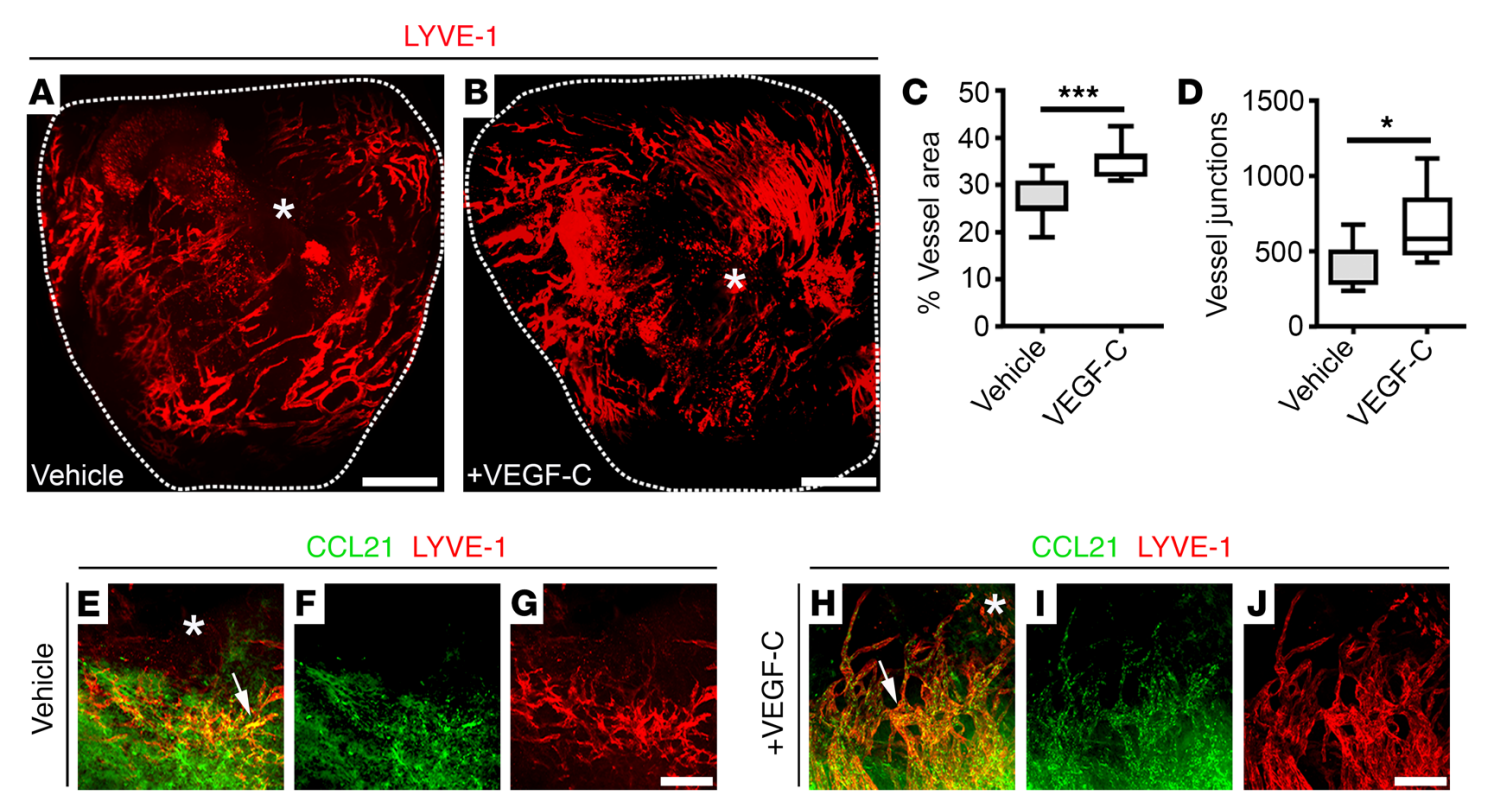
VEGF-C treatment augments cardiac lymphangiogenesis after injury. (**A** and **B**) Whole-mount immunostaining for LYVE-1 (red) to visualize the subepicardial lymphatic plexus of vehicle- and recombinant VEGF-C(C156S)–treated hearts on day 7 after MI. (**C** and **D**) Quantification of the lymphangiogenic response on day 7 after MI as percent LYVE-1^+^ lymphatic vessel area (**C**) and junction number (**D**). Data are presented as mean ± SEM; vehicle, *n* = 7 hearts; VEGF-C, *n* = 5 hearts. Significant differences were calculated using an unpaired, 2-tailed Student’s *t* test (*P ≤ 0.05, ***P ≤ 0.001). (**E**–**J**) Immunostaining for CCL21 (green) and LYVE-1 (red) in vehicle- (**E**–**G**) and recombinant VEGF-C(C156S)–treated (**H**–**J**) whole adult hearts on day 7 after MI. White arrows highlight expression of the immune cell chemoattractant cue CCL21 by lymphatic capillaries after injury. White asterisks indicate the ligating suture. Scale bars: **A** and **B**, 1 mm; **G** and **J**, 20 μm.

Initially we determined the source of VEGF-C that acts to drive the inherent lymphangiogenic response after injury. In the developing heart, the epicardium is a site of VEGF-C expression (20), and in dermal wounding studies CD11b^+^ macrophages act as a source of VEGF-C and VEGF-D in response to inflammatory stimuli (5). In the adult heart, while we found no expression of VEGF-C in the quiescent podoplanin-expressing (PDPN-expressing) epicardium or CD68^+^ tissue macrophages residing in the intact myocardium (Supplemental Figure 1, A and B; supplemental material available online with this article; https://doi.org/10.1172/JCI97192DS1), expression of VEGF-C was increased in both cell types following MI (Supplemental Figure 1, C and D), leading to substantial lymphangiogenesis and remodeling of the subepicardial lymphatic network (Figure 1A). Moreover, this response was considerably enhanced in mice treated with 100 ng/g body weight of recombinant selective VEGFR-3 agonist VEGF-C–Cys(156)Ser (21) [henceforth, VEGF-C(C156S)] on days 0, 2, 3, 4, and 6 after MI (Figure 1B) (14) and resulted in increased LYVE-1^+^ vessel area percentage (vehicle, 26.43 ± 1.82; VEGF-C, 35.28 ± 1.17; *P* ≤ 0.001) and junction number (vehicle, 378.6 ± 60.49; VEGF-C, 660.6 ± 77.31; *P* ≤ 0.05; Figure 1, C and D). Moreover, VEGF-C(C156S) treatment led to augmented expression of the leukocyte chemoattractant CCL21 by LYVE-1–expressing capillaries, indicative of an inflamed lymphatic endothelium (Figure 1, E–J). Treatment with VEGF-C(C156S) has been reported to reduce myocardial edema and pathological remodeling, leading to improved cardiac function (14, 22). However, the effect on tissue fluid constitutes an acute and relatively minor function of the responding lymphatic vasculature; consequently, we investigated whether lymphangiogenesis acts on the immune response and associated inflammation in the setting of cardiac injury.

**Figure 2:**
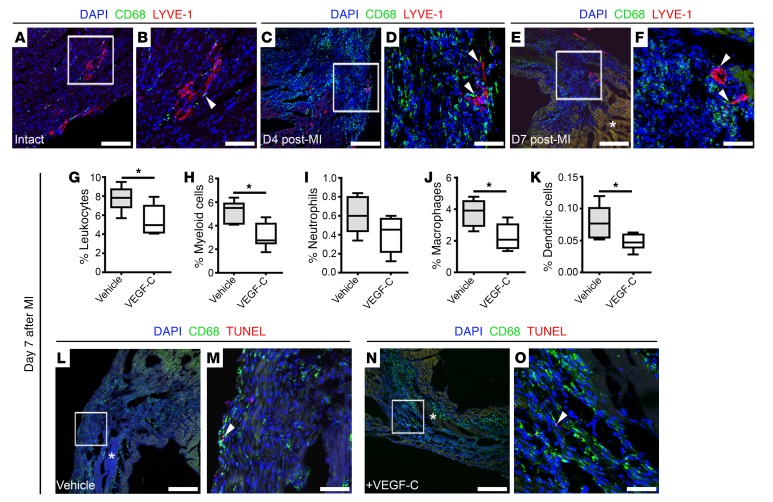
VEGF-C–driven cardiac lymphangiogenesis increases clearance of immune cells after injury. (**A**–**F**) CD68 (green) and LYVE-1 (red) immunostaining of tissue sections derived from adult intact hearts (**A** and **B**) from hearts day 4 (**C** and **D**) and day 7 (**E** and **F**) after MI, documenting close association of macrophages to lymphatic vessels (white arrowheads). (**B**) Magnified view of box shown in **A**. (**D**) Magnified view of box shown in **C**. (**F**) Magnified view of box shown in **E**. DAPI (blue) labels cell nuclei. Asterisk in **E** denotes fibrotic scarring. (**G**–**K**) Characterization of immune cell content in vehicle- and VEGF-C–treated hearts collected on day 7 after MI and analyzed by flow cytometry using antibodies against CD45 (pan-leukocyte marker), CD11b (CD45^+^CD11b^+^, myeloid cells), Ly6G (CD45^+^CD11b^+^Ly6G^+^, neutrophils), F4/80 (CD45^+^CD11b^+^Ly6G^–^F4/80^+^, macrophages), and CD11c (CD45^+^CD11b^+^Ly6G^–^F4/80^–^CD11c^+^, dendritic cells). Animals received i.p. injections of vehicle (PBS) or recombinant VEGF-C(C156S) on days 0, 2, 4, and 6 after MI. Data are presented as mean ± SEM; vehicle, *n* = 5 hearts; VEGF-C, *n* = 6 hearts. Significant differences were calculated using an unpaired, 2-tailed Student’s *t* test (*P ≤ 0.05). (**L**–**O**) CD68 (green) and TUNEL (red) immunostaining of tissue sections derived from vehicle- (**L** and **M**) and recombinant VEGF–C(C156S)–treated (**N** and **O**) hearts on day 7 after MI. (**M**) Magnified view of box shown in **L**. (**O**) Magnified view of box shown in **N**. Asterisks in **L** and **N** indicate fibrotic scarring; DAPI (blue) labels cell nuclei. White arrowheads mark rare macrophage cells undergoing apoptosis (CD68^+^TUNEL^+^). Scale bars: **A**, **C**, **E**, **L**, and **N**, 100 μm; **B**, **D**, **F**, **M**, and **O**, 20 μm.

We first probed a potential colocalization of immune cells with the expanding lymphatic plexus after MI and observed that CD68^+^ macrophages were closely associated with LYVE-1^+^ cardiac lymphatic capillaries throughout the myocardium of intact (Figure 2, A and B) and injured hearts (Figure 2, C–F). This colocalization was especially evident within the infarcted area at 4 days after MI (arrowheads in Figure 2D), the time point when a significant influx of circulating monocytes and activated macrophages undertake extensive phagocytic activity to clear neutrophils (23). Following this early acute inflammatory phase, CD68^+^ phagocytes persisted within the injured myocardium at 7 days after MI (Figure 2, E and F) — a phenomenon known to contribute to chronic inflammation (23). By contrast, in VEGF-C(C156S)–treated hearts on day 7, in which we observed enhanced lymphangiogenesis (Figure 1), we detected significantly decreased numbers of CD45^+^ leukocytes (vehicle, 7.80 ± 0.61; VEGF-C, 5.46 ± 0.67; *P* ≤ 0.05), specifically myeloid cells (CD45^+^CD11b^+^: vehicle, 5.11 ± 0.45; VEGF-C, 3.12 ± 0.44; *P* ≤ 0.05) and myeloid subtypes such as CD45^+^CD11b^+^Ly6G^–^F4/80^+^ macrophages (vehicle, 3.76 ± 0.40; VEGF-C, 2.24 ± 0.34; *P* ≤ 0.05) and CD45^+^CD11b^+^Ly6G^–^F4/80^–^CD11c^+^ dendritic cells (vehicle, 0.078 ± 0.012; VEGF-C, 0.048 ± 0.005; *P* ≤ 0.05), as determined by multi-marker flow cytometry (Figure 2, G–K, and Supplemental Figure 2, A–H). Importantly, TUNEL staining revealed scarce apoptotic CD68^+^ immune cells (ranging from 0 to 2 CD68^+^TUNEL^+^ cells per tissue section) in both vehicle- and VEGF-C(C156S)–treated hearts on day 7 after MI (Figure 2, L–O), indicating that the effect of VEGF-C treatment was not due to increased apoptosis triggered by factors transported through the expanded lymphatic network.

To address the possibility that the reduced immune cell content was independent of lymphangiogenesis, we performed flow cytometry (Supplemental Figure 2, A–H) on tissue derived from vehicle- and VEGF-C(C156S)–treated hearts collected on days 1 and 4 after MI, before any notable evidence of lymphatic sprouting (Supplemental Figure 3). No significant differences in immune cell numbers were observed between vehicle- and VEGF-C(C156S)–treated hearts on day 1 (Supplemental Figure 3, A–E) or day 4 (Supplemental Figure 3, F–J) after MI, suggesting that there was no substantial early effect of VEGF-C(C156S) treatment on cardiac lymphatic vessel permeability to enable clearance of leukocytes from the injured heart. Instead, clearance was only evident by day 7 after MI following the establishment of an extensive lymphatic network draining the infarcted area (Figure 1), supporting the hypothesis that lymphangiogenesis augments the resolution of inflammation after injury.

To further characterize the infarcted milieu following VEGF-C(C156S) treatment, we investigated the expression of key macrophage markers and cytokines (24), including the costimulatory receptors CD80 and CD86, which are enriched in so-called proinflammatory M1 subtype macrophages, and CD206 and Chi3l3, markers of the pro-repair M2 subtype macrophages; proinflammatory cytokines IL-1β, IL-6, and TNF-α; the antiinflammatory cytokine IL-10; and profibrotic factor TGF-β1 (24). No significant differences in expression of any of these factors were observed between vehicle- and VEGF-C(C156S)–treated hearts on day 7 after MI (Supplemental Figure 4), suggesting that lymphangiogenesis driven by VEGF-C(C156S) treatment does not alter macrophage polarization or favor a specific M1 or M2 subtype, but instead serves to reduce the overall macrophage load in the injured heart.

### LYVE-1 is required for immune cell clearance after MI.

**Figure 3:**
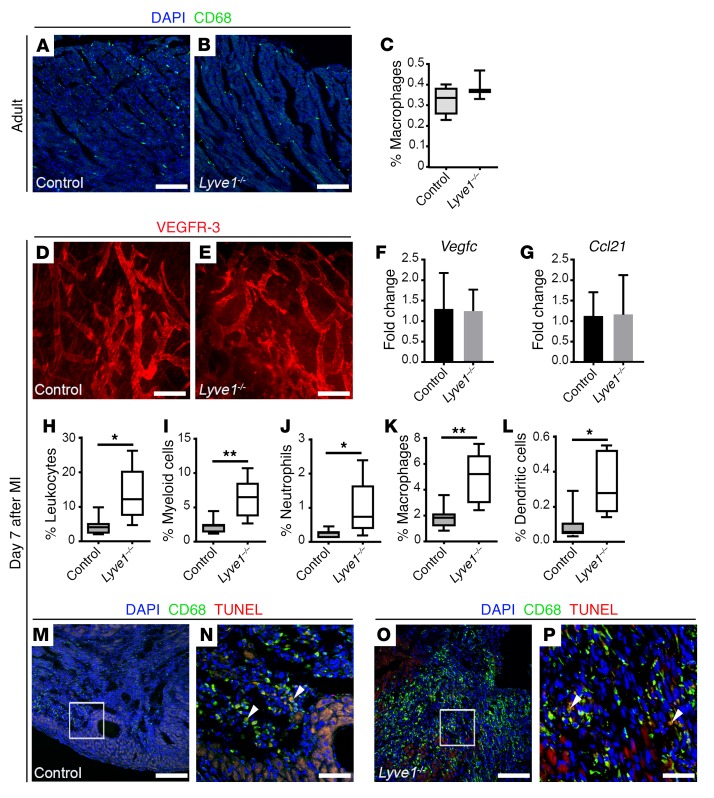
LYVE-1 is required for immune cell clearance after MI. (**A** and **B**) CD68 (green) immunostaining of sections derived from control and *Lyve1^–/–^* intact hearts, documenting the presence of resident macrophages throughout the myocardium of mutant hearts, compared with controls. DAPI (blue) labels cell nuclei. (**C**) Quantification of resident macrophages (CD45^+^CD11b^+^Ly6G^–^F4/80^+^) in the intact adult heart by flow cytometry. Data are presented as mean ± SEM; control, *n* = 4 hearts; *Lyve1^–/–^*, *n* = 4 hearts. No significant differences were observed (unpaired, 2-tailed Student’s *t* test). (**D** and **E**) Whole-mount immunostaining for VEGFR-3 (red) revealing comparable superficial lymphatic networks with lymphangiogenic capillary tips in control and *Lyve1^–/–^* hearts on day 7 after MI. (**F** and **G**) *Vegfc* and *Ccl21* expression analysis by qRT-PCR showing no differences in expression levels in control and *Lyve1^–/–^* hearts on day 7 after MI. Data are presented as mean ± SEM; control, *n* = 4 hearts; *Lyve1^–/–^*, *n* = 6 hearts. (**H**–**L**) Characterization of the immune cell content in control and *Lyve1^–/–^* hearts collected on day 7 after MI and analyzed by flow cytometry using antibodies against CD45 (pan-leukocyte marker), CD11b (CD45^+^CD11b^+^, myeloid cells), Ly6G (CD45^+^CD11b^+^Ly6G^+^, neutrophils), F4/80 (CD45^+^CD11b^+^Ly6G^–^F4/80^+^, macrophages), and CD11c (CD45^+^CD11b^+^Ly6G^–^F4/80^–^CD11c^+^, dendritic cells). Data are presented as mean ± SEM; control, *n* = 7 hearts; *Lyve1^–/–^*, *n* = 5 hearts. Significant differences were calculated using an unpaired, 2-tailed Student’s *t* test (*P ≤ 0.05, **P ≤ 0.01). (**M**–**P**) CD68 (green) and TUNEL (red) immunostaining of sections derived from control (**M** and **N**) and *Lyve1^–/–^* (**O** and **P**) hearts on day 7 after MI. (**N** and **P**) Magnified views of boxes shown in **M** and **O**. DAPI (blue) labels cell nuclei. White arrowheads mark rare macrophages undergoing apoptosis (CD68^+^TUNEL^+^). Note increased CD68 expression in *Lyve1^–/–^* compared with controls. Scale bars: **A**, **B**, **M**, and **O**, 100 μm; **D** and **E**, 200 μm; **N** and **P**, 20 μm.

To further test the hypothesis that immune cell clearance downstream of lymphangiogenesis improves cardiac outcome after MI, we utilized *Lyve1^–/–^* constitutive KO mice (25). LYVE-1 is a homolog of the CD44 glycoprotein that is highly expressed in the overlapping junctions of lymphatic capillaries (26). LYVE-1 is dispensable during lymphatic development (25) but is required for leukocyte docking, whereby both macrophages and dendritic cells have been shown to present HA to engage LYVE-1, facilitating their adhesion to, and transit across, lymphatic endothelium for trafficking under inflammatory conditions (19, 27). LYVE-1 is also an early marker of yolk sac–derived macrophages that populate the heart during embryonic development (28); however, we observed comparable representation of CD68^+^ tissue macrophages residing in the intact adult myocardium in LYVE-1–deficient mice and littermate controls, suggesting no initial differences in macrophage numbers at baseline (Figure 3, A–C). Following injury *Lyve1^–/–^* mutants exhibited a left ventricular ischemic area at risk (LV AAR; Supplemental Figure 5) equivalent to that of controls and displayed a grossly normal cardiac lymphangiogenic response following MI, comprising equivalent expansion of the VEGFR-3–expressing lymphatic capillary network (Figure 3, D and E), and expression of the lymphangiogenic factor VEGF-C (Figure 3F) and leukocyte chemoattractant cue CCL21 (Figure 3G; also Supplemental Figure 6, A–C). The grossly normal lymphangiogenic response in *Lyve1^–/–^* mice was further supported by comparable upregulation of genes encoding key regulators of lymphatic vessel expansion (29), such as PROX1 and VEGFR-3 (Supplemental Figure 6, D–F). Likewise, quantitative real-time PCR (qRT-PCR) revealed comparable expression levels of *Has2* mRNA, encoding the main HA synthase of murine leukocytes (30), in LYVE-1–deficient and control hearts on day 7 after MI (Supplemental Figure 6G).

We next investigated immune cell clearance in *Lyve1^–/–^* hearts after MI by flow cytometry and observed significantly increased numbers of CD45^+^ leukocytes (control, 4.42% ± 1.02%; *Lyve1^–/–^*, 13.54% ± 3.58%; *P* ≤ 0.05), specifically myeloid cells (CD45^+^CD11b^+^: control, 2.27% ± 0.42%; *Lyve1^–/–^*, 6.21% ± 1.33%; *P* ≤ 0.01) and myeloid subtypes such as CD45^+^CD11b^+^Ly6G^+^ neutrophils (control, 0.25% ± 0.04%; *Lyve1^–/–^*, 0.96% ± 0.38%; *P* ≤ 0.05), CD45^+^CD11b^+^Ly6G^–^F4/80^+^ macrophages (control, 1.83% ± 0.34%; *Lyve1^–/–^*, 4.90% ± 0.90%; *P* ≤ 0.01) and CD45^+^CD11b^+^Ly6G^–^F4/80^–^CD11c^+^ dendritic cells (control, 0.098% ± 0.034%; *Lyve1^–/–^*, 0.334% ± 0.081%; *P* ≤ 0.05) retained in the heart (Figure 3, H–L, and Supplemental Figure 2, A–H). Importantly, immunostaining revealed an accumulation of CD68^+^ macrophages within the infarcted area in mutant hearts compared with controls by day 7 after MI, but with an equivalent low incidence of apoptotic CD68^+^ cells (typically 0–2 CD68^+^TUNEL^+^ cells per tissue section; Figure 3, M–P). Despite the macrophage accumulation, there were no significant differences in expression of proinflammatory/pro-reparative macrophage subtype markers and cytokines in mutant hearts on day 7 after MI, when compared with controls (Supplemental Figure 7), as observed for the reciprocal gain-of-function VEGF-C(C156S)–treated hearts (Supplemental Figure 4), suggesting that the retained immune cell population was not enriched for a specific macrophage subtype.

### Cardiac immune cells are cleared to MLNs after MI.

**Figure 4:**
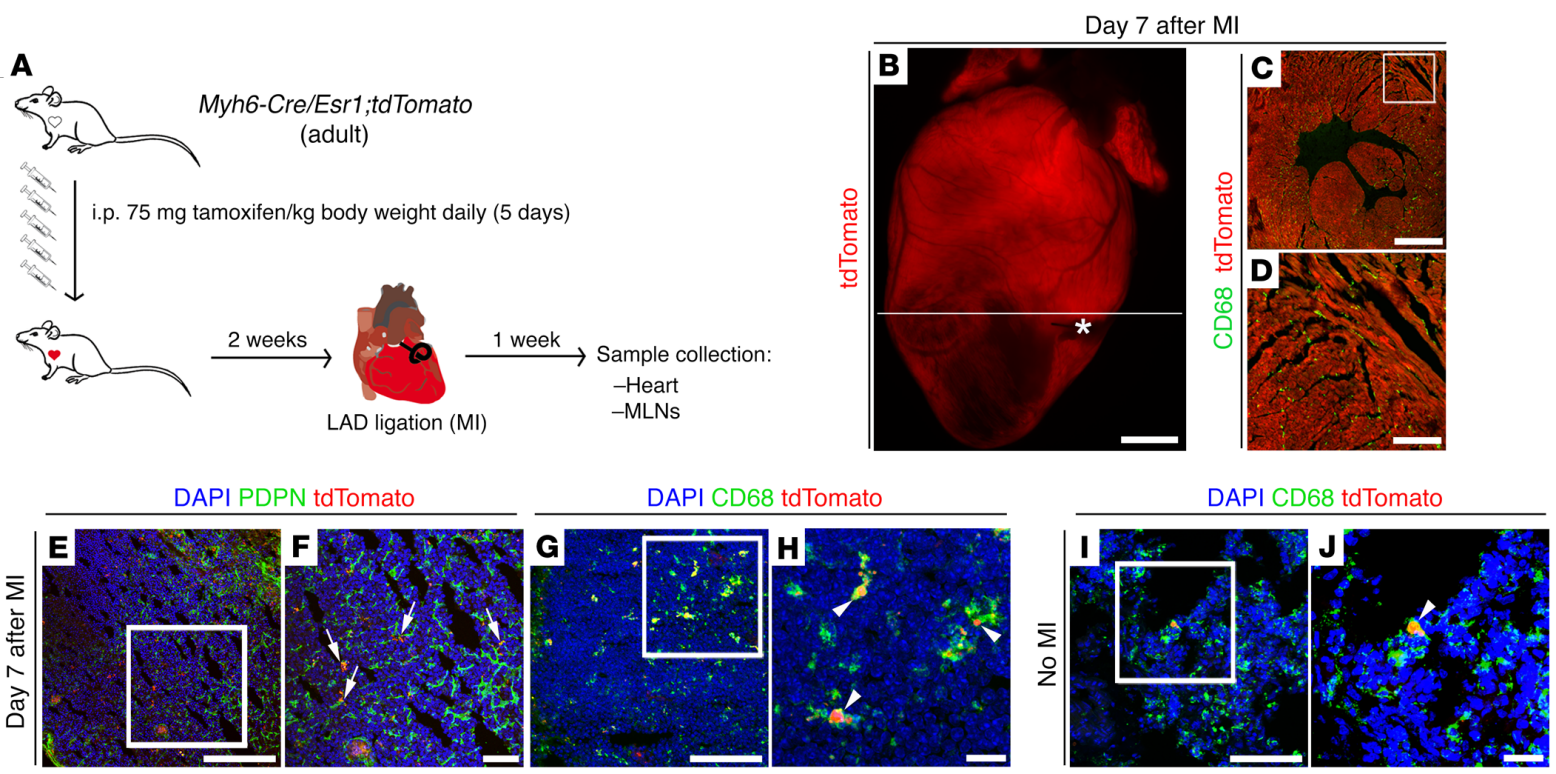
Cardiac immune cells are cleared to MLNs after injury. (**A**) Schematic of tamoxifen-induced labeling of adult cardiomyocytes in *Myh6-Cre/Esr1;tdTomato* mice to probe phagocytic cell trafficking to MLNs via the cardiac lymphatic system on day 7 after injury. (**B**–**D**) Visualization of endogenous tdTomato (red) fluorescence alone or in combination with CD68 (green) immunostaining (macrophage marker), documenting efficient labeling of cardiomyocytes in the adult heart. White asterisk marks the ligating suture; white line marks the plane of sectioning of the whole heart. Note that the section in **C** is derived from the heart in **B**, and **D** is a magnified view of white box in **C**. (**E** and **F**) PDPN (green) immunostaining and tdTomato fluorescence marking red-labeled particles in close association with PDPN-expressing lymphatic capillaries (white arrows) within MLNs of tamoxifen-induced *Myh6-Cre/Esr1;tdTomato* mice at 7 days after MI. (**G**–**J**) CD68 (green) immunostaining combined with tdTomato (red) fluorescence indicating that red particles are contained within CD68^+^ phagocytic cells (white arrowheads). The CD68^+^/tdTomato-labeled cell population is increased in MLNs after MI (compare **G** and **H** with **I** and **J**). (**F**, **H**, and **J**) Magnified views of white boxes in **E**, **G**, and **I**. DAPI (blue) labels cell nuclei. Scale bars: **C**, **E**, **G**, and **I**, 100 μm; **B**, 1 mm; **D**, 300 μm; **F**, 50 μm; **H** and **J**, 20 μm.

Collectively, our data reveal a role for cardiac lymphatics in the resolution of inflammation after MI, in a process dependent on LYVE-1 and augmented by VEGF-C(C156S)–induced lymphangiogenesis. Moreover, active clearance, via lymph rather than in situ apoptosis of immune cells is likely to be the primary mechanism for inflammation resolution in this setting. In order to investigate this further, we focused our attention on the MLNs as the predicted sites for cardiac lymphatic drainage based on their anatomical location in relation to the heart (9). To confirm MLNs as the secondary lymphatic organs serving the heart, we employed a mouse model expressing a tdTomato reporter in the α–myosin heavy chain–positive (αMHC-positive) adult myocardium (*Myh6-Cre/Esr1;tdTomato*; Figure 4, A–D). Following MI and tamoxifen induction of the reporter, tdTomato-positive particles were detected within CD68^+^ cells in MLNs adjacent to PDPN-expressing lymphatic sinuses (Figure 4, E–H), indicative of phagocytosis of labeled myocyte debris (tdTomato^+^ myosin fragments) and subsequent transport via afferent cardiac lymphatics. In contrast, CD68^+^/tdTomato^+^ macrophages were rarely observed in MLNs of intact animals (no MI; Figure 4, I and J).

**Figure 5:**
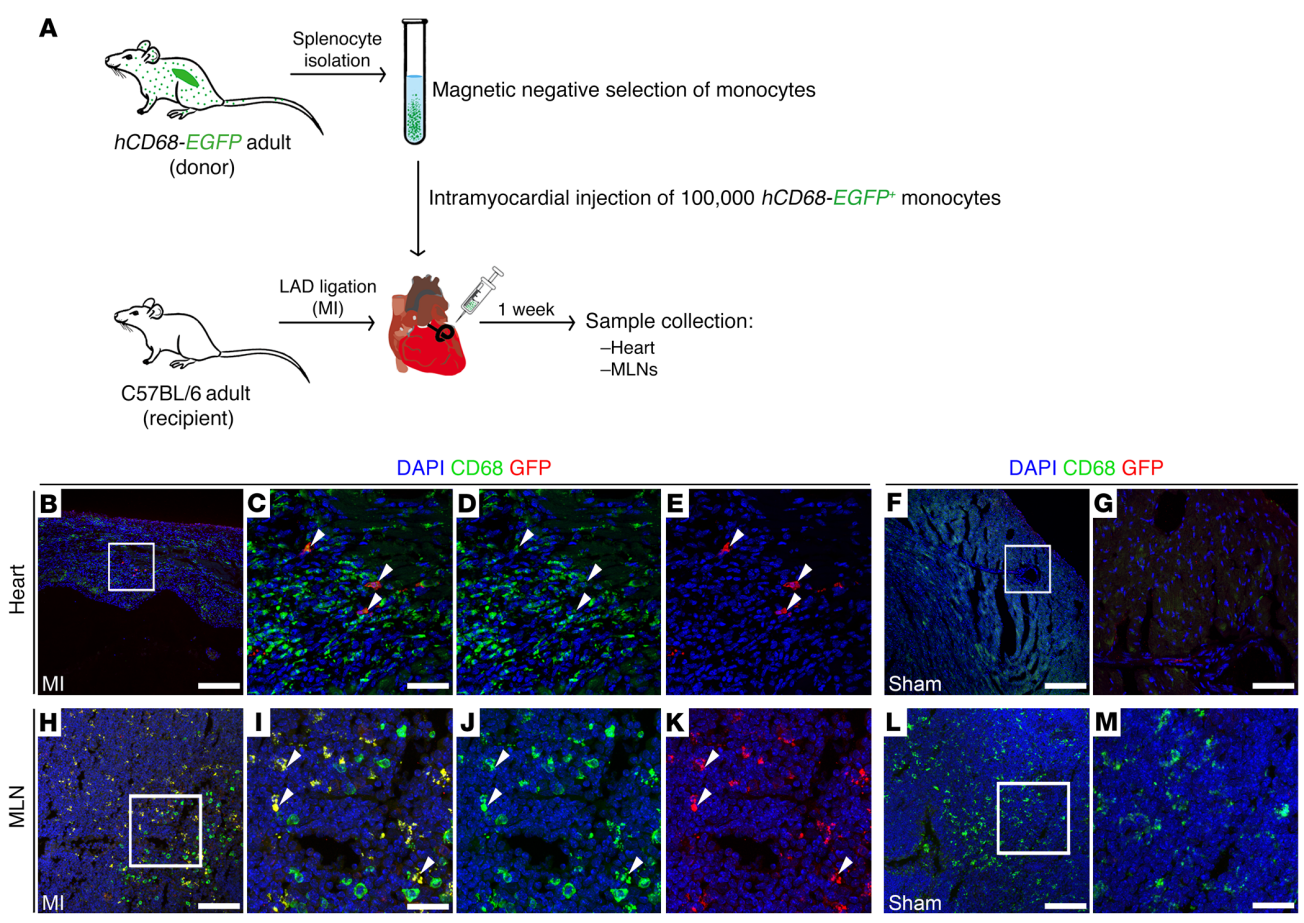
Adoptive transfer of splenic GFP^+^ monocytes confirms immune cell clearance to MLNs after MI. (**A**) Schematic of the adoptive cell transfer approach using hCD68-EGFP transgenic mice as splenic GFP^+^ monocyte donor and recipient C57BL/6 adult mice receiving intramyocardial delivery of labeled monocytes at the time of LAD ligation, to assess immune cell trafficking to MLNs. (**B**–**G**) GFP (red) and CD68 (green) immunostaining (macrophage marker) of tissue sections documenting engraftment of CD68^+^GFP^+^ monocytes within the injury area at 7 days after MI (white arrowheads). No GFP-labeled cells were detected in sham-operated animals (**F** and **G**). (**C**–**E**) Magnified views of box shown in **B**. (**G**) Magnified view of box shown in **F**. DAPI (blue) labels cell nuclei. (**H**–**M**) GFP (red) and CD68 (green) immunostaining of tissue sections derived from MLNs of MI (**H**–**K**) and sham-operated (**L**–**M**) animals, indicating the presence of cleared CD68^+^GFP^+^ phagocytic cells (white arrowheads) in MLNs after MI. (**I**, **J**, and **K**) Magnified views of box in **H**. (**M**) Magnified view of box in **L**. DAPI (blue) labels cell nuclei. Scale bars: 100 μm; except **C**, **G**, **I**, and **M**, 20 μm.

The detection of tdTomato^+^ myosin fragments in CD68^+^ cells within MLNs after MI could potentially result from clearance of labeled debris via lymph, and phagocytosis by MLN-resident macrophages. To unequivocally confirm trafficking of cardiac immune cells to MLNs, we therefore employed adoptive cell transfer using an *hCD68-EGFP* transgenic mouse line (Figure 5). Specifically, splenic GFP^+^ monocytes were isolated and transferred to a recipient WT mouse, via intramyocardial delivery, at the time of coronary artery ligation (Figure 5A). CD68^+^GFP^+^ cells were observed within the infarcted milieu at 7 days after MI, but not in sham-operated hearts (Figure 5, B–G), indicative of engraftment of the transferred cells and their mobilization to the injury area. Remarkably, CD68^+^GFP^+^ cells were also detected in draining MLNs of animals undergoing adoptive cell transfer and MI (Figure 5, H–M), further supporting the hypothesis that immune cells are cleared via afferent cardiac lymphatics.

**Figure 6:**
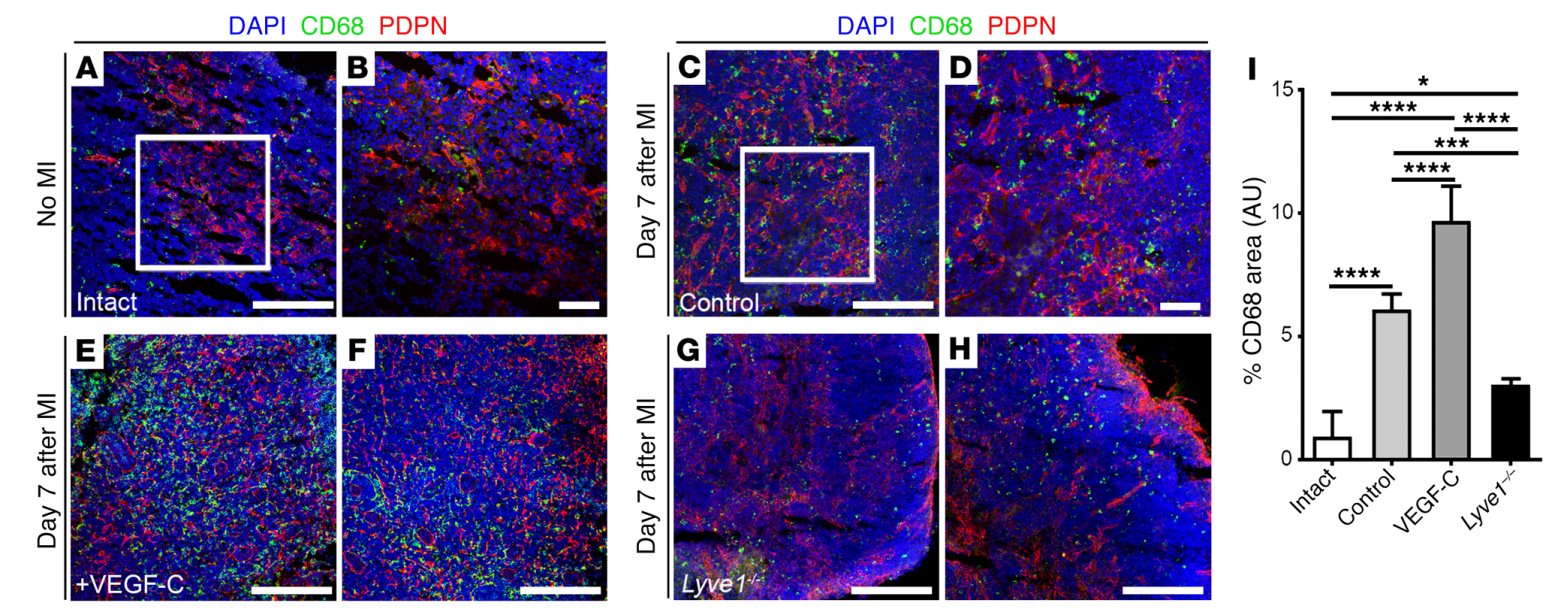
VEGF-C–driven cardiac lymphangiogenesis promotes clearance of immune cells to MLNs after injury. (**A**–**H**) CD68 (green) and PDPN (red) immunostaining of tissue sections derived from intact (no MI; **A** and **B**), control (**C** and **D**), recombinant VEGF-C(C156S)–treated (**E** and **F**), and *Lyve1^–/–^* (**G** and **H**) MLNs collected 7 days after MI. (**B** and **D**) Magnified views of white boxes in **A** and **C**. (**E** and **F**) Representative views of 2 different VEGF-C–treated MLNs. (**G** and **H**) Representative views of 2 different *Lyve1^–/–^* MLNs. Note the relative abundance of CD68^+^ macrophages in VEGF-C–treated MLNs, compared with *Lyve1^–/–^* (compare **E** and **F** with **G** and **H**). DAPI (blue) labels cell nuclei. (**I**) Quantification of macrophage proportion as percent CD68^+^ total staining area/total DAPI-labeled tissue area × 100. Data are presented as mean ± SEM; intact, *n* = 4 MLNs; control, *n* = 8 MLNs; VEGF-C, *n* = 4 MLNs; *Lyve1^–/–^*, *n* = 4 MLNs. Note that 1 MLN was analyzed per mouse. Significant differences were calculated using 1-way ANOVA followed by Tukey’s multiple comparisons test (*P ≤ 0.05 for intact vs. *Lyve1^–/–^*; ***P ≤ 0.001 for control vs. *Lyve1^–/–^*; and ****P ≤ 0.0001 for intact vs. control, control vs. VEGF-C, intact vs. VEGF-C, and VEGF-C vs. *Lyve1^–/–^*). Scale bars: 100 μm; except **B** and **D**, 50 μm.

We next analyzed MLNs dissected from VEGF-C(C156S)–treated and *Lyve1^–/–^* animals on day 7 following MI (Figure 6, A–I). While in control/vehicle-treated animals there was an increase in CD68^+^ macrophages (Figure 6, C and D) above baseline (intact/no MI; Figure 6, A and B), the increase in phagocyte number was significantly higher in MLNs of VEGF-C(C156S)–treated animals (Figure 6, E, F, and I; intact: 0.96% ± 0.49%; control: 6.09% ± 0.23%; VEGF-C: 9.69% ± 0.71%; *P* ≤ 0.0001 [intact vs. control], *P* ≤ 0.0001 [intact vs. VEGF-C], and *P* ≤ 0.0001 [control vs. VEGF-C]). In contrast, MLNs derived from *Lyve1^–/–^* animals revealed significantly lower CD68^+^ cell content, which was at a level more similar to baseline (intact) than control/vehicle-treated after MI (Figure 6, G–I; *Lyve1^–/–^*: 3.05% ± 0.13%; *P* ≤ 0.05 [intact vs. *Lyve1^–/–^*]; *P* ≤ 0.001 [control vs. *Lyve1^–/–^*]; and *P* ≤ 0.0001 [VEGF-C vs. *Lyve1^–/–^*]). Of significance, no CD68^+^ phospho–histone H3^+^ (CD68^+^PH3^+^) phagocytic cells were found in MLNs from VEGF-C(C156S)–treated animals, akin to control/vehicle-treated and *Lyve1^–/–^* MLNs (Supplemental Figure 8), thus excluding the possibility that the augmented CD68^+^ cell population (Figure 6, E, F, and I) was due to a proliferative response of lymph node–resident macrophages to the VEGF-C(C156S) treatment.

To determine whether increasing lymphangiogenesis may rescue the phenotype observed in *Lyve1^–/–^* animals, we treated KO animals with VEGF-C(C156S) (Supplemental Figure 9). No significant differences in immune cell numbers were observed between vehicle- and VEGF-C(C156S)–treated *Lyve1^–/–^* hearts (Supplemental Figure 9, A–E) or MLNs (Supplemental Figure 9, F–J) on day 7 after MI, suggesting that VEGF-C treatment does not alter trafficking in the mutants as it does in control hearts (Figure 2). These findings reinforce the idea that *Lyve1^–/–^* cardiac lymphatics have impaired immune cell uptake, which prevents trafficking, irrespective of lymphangiogenesis. Together, these data support clearance of immune cells via lymph into draining MLNs, a process enhanced by VEGF-C–induced cardiac lymphangiogenesis and dependent on LYVE-1.

### Disruption of LYVE-1–dependent clearance of immune cells by lymphatics is detrimental to cardiac function after MI.

**Figure 7:**
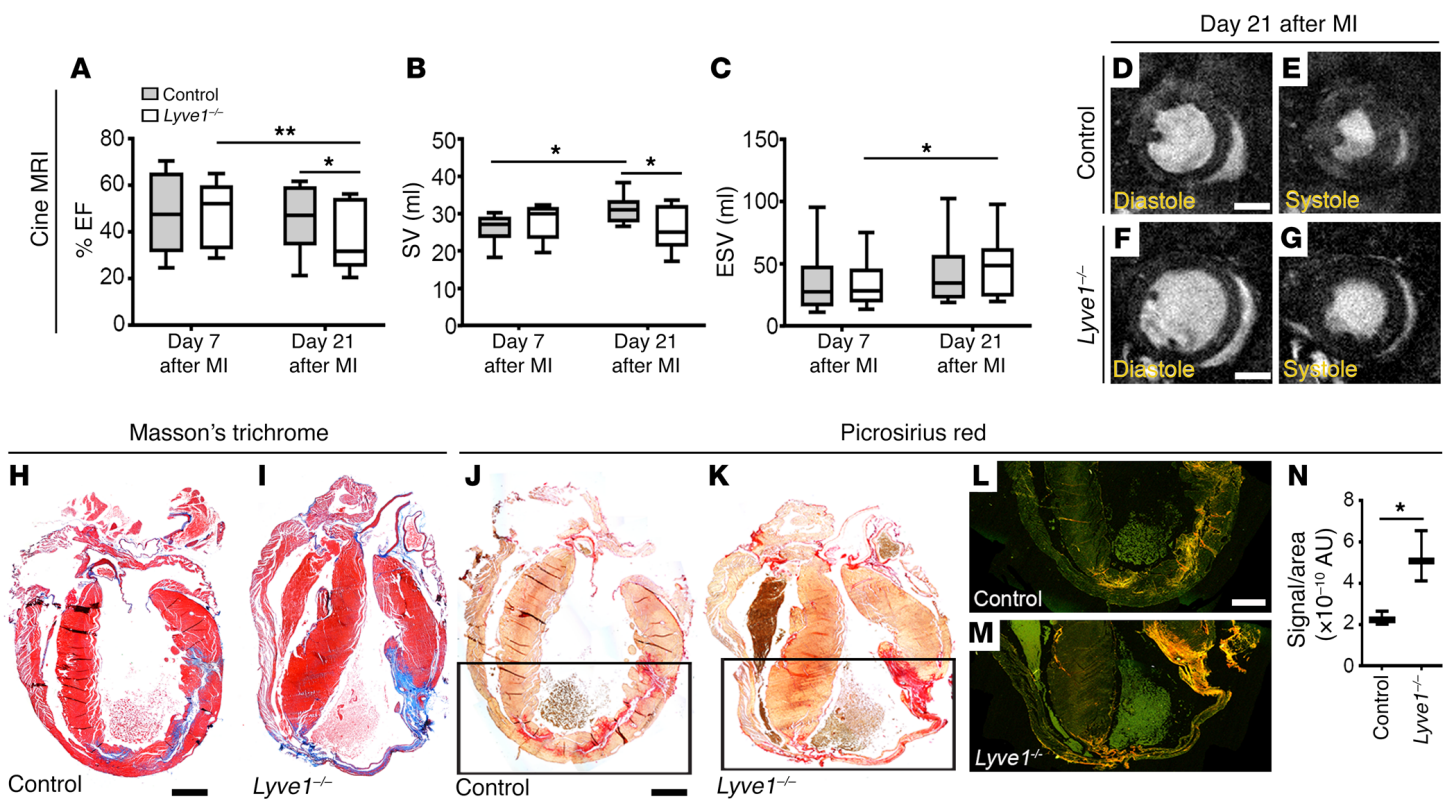
Disruption of LYVE-1–dependent clearance of immune cells by lymphatics is detrimental to cardiac function after injury. (**A**–**C**) Longitudinal cine MRI analyses of infarcted control and *Lyve1^–/–^* hearts on days 7 and 21 after injury showing reduced EF (**A**), SV (**B**), and ESV (**C**) in mutants, compared with controls. Data are presented as mean ± SEM; control, *n* = 10 hearts; *Lyve1^–/–^* , *n* = 10 hearts. Significant differences were calculated using 2-way ANOVA with repeated measures (*P ≤ 0.05, **P ≤ 0.01). (**D**–**G**) Representative 1-mm-thick mid-ventricular short-axis cine MRI frames for control (**D** and **E**) and *Lyve1^–/–^* (**F** and **G**) hearts in diastole (**D** and **F**) and systole (**E** and **G**) on day 21 after MI. (**H**–**N**) Histological characterization of control and *Lyve1^–/–^* hearts on day 21 after MI using Masson’s trichrome (**H** and **I**) and Picrosirius red staining (**J**–**N**), documenting excessive collagen deposition/fibrotic scarring (blue in **H** and **I**; red in **J** and **K**; yellow-orange birefringence in **L** and **M**) in mutant hearts. Note that Picrosirius staining was visualized under brightfield (**J** and **K**) and polarized light (**L** and **M**), leading to birefringence of the collagen fibers, to further characterize the type of fibers making up the scar, i.e. type I (thicker; yellow-orange) or type III (thin; green). (**N**) Quantification of fibrotic scarring as (Picrosirius) yellow-orange birefringence signal/area ratio. Data are presented as mean ± SEM; control, *n* = 5 hearts; *Lyve1^–/–^*, *n* = 5 hearts. Significant differences were calculated using an unpaired, 2-tailed Student’s *t* test (*P ≤ 0.05). Scale bars: 1 mm; except **D** and **F**, 2 mm.

To investigate whether immune cell accumulation (failed clearance) in *Lyve1*-deficient mice is detrimental to cardiac function, we performed a longitudinal cine MRI study with mice scanned on days 7 and 21 after MI (Figure 7, A–G, Supplemental Table 1, and Supplemental Videos 1 and 2). These time points were selected based on our observations of active lymphangiogenesis and immune cell trafficking to regional MLNs by day 7 (Figures 1–6) and functional improvement, as previously determined in VEGF-C(C156S)–treated hearts, by day 21 (14). MRI revealed that both percentage LV ejection fraction (EF) and stroke volume (SV), calculated as the difference between end-diastolic volume (EDV) and end-systolic volume (ESV) (31), were significantly reduced in *Lyve1^–/–^* mice compared with controls (Figure 7, A and B, and Supplemental Table 1; EF: day 7 control, 46.91% ± 5.15%, *Lyve1^–/–^*, 47.98% ± 4.48%; day 21 control, 45.86% ± 4.26%, *Lyve1^–/–^*, 38.45% ± 4.69%; *P* ≤ 0.01 [day 7 vs. 21 *Lyve1^–/–^*] and *P* ≤ 0.05 [day 21 control vs. day 21 *Lyve1^–/–^*]; SV: day 7 control, 26.00% ± 1.25%, *Lyve1^–/–^*, 27.85% ± 1.43%; day 21 control, 30.93% ± 1.15%, *Lyve1^–/–^*, 25.52% ± 1.84%; *P* ≤ 0.05 [day 7 vs. 21 control] and *P* ≤ 0.05 [day 21 control vs. day 21 *Lyve1^–/–^*]). Moreover, the ESV in *Lyve1*-mutant hearts was significantly increased (Figure 7, C–G, and Supplemental Table 1; day 7 control, 36.47% ± 8.26%, *Lyve1^–/–^*, 34.34% ± 6.17%; day 21 control, 43.33% ± 9.00%, *Lyve-1^–/–^*, 47.18% ± 7.92%; *P* ≤ 0.05; day 7 vs. 21 *Lyve1^–/–^*), and there was evidence of LV wall thinning (Supplemental Videos 1 and 2), which is known to contribute to a decline in cardiac function after MI. Histological analyses by Masson’s trichrome and Picrosirius red staining revealed extensive collagen deposition and scarring in *Lyve1^–/–^* hearts on day 21 after MI compared with controls (Picrosirius signal/area ratio quantification: control, 2.28 × 10^–10^ ± 1.79 × 10^–11^; *Lyve1^–/–^*, 5.22 × 10^–10^ ± 6.99 × 10^–11^; *P* ≤ 0.05; Figure 7, H–N). Thus, in the absence of any gross lymphatic vessel defects at baseline or abnormal lymphangiogenic response after MI, these data collectively suggest that impaired immune cell uptake and clearance in *Lyve1* mutants resulted in a progressive deterioration in heart function, accompanied by elevated pathological remodeling and fibrosis.

## Discussion

Taken together, our results demonstrate an essential immunomodulatory role for lymphangiogenesis in regulating heart function. We reveal that stimulation of lymphangiogenesis by VEGF-C treatment after heart injury leads to early resolution of inflammation through enhanced immune cell clearance, in a process dependent on the lymphatic receptor LYVE-1 (Figures 1–3). While we report direct effects on innate immune cell clearance, the improved outcome may also be mediated, at least in part, via the adaptive immune response and the recruitment of lymphocyte subpopulations that act as negative regulators of inflammation (32). Tregs infiltrate the infarcted tissue as a third wave of (adaptive) immune cells, and promote differentiation of macrophages to an antiinflammatory phenotype and modulate protease synthesis by cardiac fibroblasts to aid wound healing and repair (33, 34). It is feasible that the increased clearance of dendritic cells we observed following VEGF-C treatment (Figure 2K) enhances the programing of remote naive T cells (35) residing in draining lymph nodes to Tregs, which when mobilized into the injured heart alter macrophage polarization to favor a more antiinflammatory/pro-reparative M2 state (reviewed in ref. 27). However, our expression analyses revealed no evidence of alterations in macrophage polarization, M1/M2 marker ratios, cytokine profiles, or selection of specific macrophage subtypes in the infarcted milieu at 7 days after MI (Supplemental Figure 4), arguing against an indirect pro-reparative effect via the adaptive immune system.

Our study suggests that macrophage load correlates directly with effective cardiac repair and improved functional outcome. This is consistent with the fact that splenectomized or monocyte-depleted mice and human patients on steroids adversely lowering monocyte numbers (36, 37) — as well as patients with elevated monocytosis (increased monocyte load) and apoE-null mice with increased monocytes (38, 39) — have impaired peripheral wound healing. Thus, achieving the optimal monocyte/macrophage loading after injury in general terms represents a therapeutic opportunity (reviewed in ref. 40) that in the heart may be achieved by targeting the cardiac lymphatic system to spatiotemporally constrain the innate immune response. By efficiently clearing immune cells away from the injury site to MLNs, the lymphatic network dampens the acute immune response, preventing its long-term escalation to a more chronic inflammatory setting, which in turn favors more optimal tissue repair and a beneficial outcome after MI. Therefore, therapeutic strategies to invoke lymphangiogenesis may prevent the inflammation-dependent progression to heart failure in acute MI patients with ischemic heart disease.

## Methods

### Animal models.

Mice were housed and maintained in a controlled environment by University of Oxford Biomedical Services. *Lyve1^–/–^*, *hCD68-EGFP*, and *R26R^tdTomato^* mice were kept on a C57BL/6J inbred background and have been described previously (25, 36, 41). *Myh6-Cre/Esr1* mice were purchased from the Jackson Laboratory and kept on a C57BL/6J inbred background. For experiments where WT mice were required, C57BL/6J animals were used (Charles River Laboratories). For tamoxifen-dependent tissue-specific gene activation using the *Myh6-Cre/Esr1* driver, 2 mg tamoxifen (Sigma-Aldrich) was administered daily (for 5 days) to 8- to 10-week-old *Myh6-Cre/Esr1;tdTomato* mice by i.p. injection. There was a 2-week waiting period between the last i.p. injection of tamoxifen and the surgical procedure to induce MI.

### MI.

All surgical and pharmacological procedures were performed in accordance with the Animals (Scientific Procedures) Act 1986 (Home Office, United Kingdom). C57BL/6 WT, *Lyve1^–/–^*, and tamoxifen-treated *Myh6-Cre/Esr1*;*R26R^tdTomato^* adult mice (10–14 weeks old; 25–30 g body weight) were used in MI experiments. MI was induced in isoflurane-anesthetized mice by permanent ligation of the left anterior descending coronary artery (LAD). For sham controls, a suture was passed under the LAD but not ligated. Upon recovery (day 0), C57BL/6 WT animals were administered 100 ng/g body weight of recombinant VEGF-C–Cys(156)Ser (R&D Systems) or vehicle (PBS) i.p. on days 0, 2, 3, 4, and 6 after MI. Hearts were harvested at 7 days following LAD ligation (day 7 after MI). For the day 1 after MI experimental group, a single i.p. injection of vehicle (PBS) or recombinant VEGF-C(C156S) was given at recovery from surgery (day 0), and hearts were collected at 1 day following ligation. For the day 4 after MI experimental group, animals received i.p. injections of vehicle (PBS) or recombinant VEGF-C(C156S) on days 0, 2, and 3 after MI, and hearts were harvested at 4 days following ligation. Hearts and MLNs collected for immunostaining were washed in ice-cold PBS prior to overnight fixation in a 4% formaldehyde solution at 4°C. Hearts for flow cytometry were washed in ice-cold HBSS (Life Technologies) prior to tissue digestion. Hearts for subsequent RNA extraction were removed and immediately placed in a CryoTube (Nunc, Thermo Fisher Scientific), which was submerged in liquid nitrogen. For RNA extraction, post-MI hearts were dissected into infarct and remote regions before snap freezing.

### Assessment of area at risk.

Following LAD ligation of C57BL/6 WT and *Lyve1^–/–^* mice, Evans Blue dye (1 ml of 2% Evans Blue in distilled water) was perfused through the jugular vein to demarcate the area of non-perfused myocardium due to occlusion of the coronary artery (blue, perfused; pink/red, non-perfused). Hearts were rapidly extracted and frozen at –20°C for 2 hours to facilitate manual slicing of 6 transverse slices of approximately 1-mm thickness from apex to the base of the ventricles. Heart slices were briefly incubated in 2% formaldehyde solution at room temperature, washed in PBS, blotted dry, weighed, and imaged using a Zeiss Axio Imager Z1 microscope with an AxioCam MRm camera attachment running AxioVision software release 4.8 (Zeiss). Quantifications were performed on transverse slices of the LV using Fiji software (NIH Image), and area at risk (AAR) was expressed as a fraction of LV area (AAR/LV %).

### Immunodetection methods.

Whole-mount hearts or cryosections (hearts and MLNs) were processed for indirect immunofluorescence using standard methods. Primary antibodies are listed in Supplemental Table 2. Alexa Fluor secondary antibodies (Invitrogen, 1:200) were used in all cases. Imaging was performed using a Zeiss Z1 Light sheet microscope (whole-mount tissue) or an Olympus FV1000 confocal microscope (tissue sections/whole-mount tissues). Images were digitally captured and processed using Fiji software. Analysis of lymphatic vessel area and junction number calculations were performed using AngioTool software (37).

### Single-cell isolation of cardiac cells.

Post-MI hearts harvested for flow cytometry studies were isolated, placed in cold HBSS (Life Technologies), finely minced into small pieces, and digested with collagenase type II (Worthington Biochemical Corp.) solution (containing 500 U/ml HBSS) at 37°C for 30 minutes with agitation. Supernatant was removed and 10% heat-inactivated FBS (Sigma-Aldrich) added. The remaining tissue was digested with a fresh collagenase solution for a total of 3 times. Cell suspensions were combined and filtered through a 40-μm cell strainer (BD Falcon). Cells were centrifuged and washed with PBS, and Red Cell Lysis buffer (BioLegend) was used according to the manufacturer’s instructions to remove red blood cells. Isolated single cardiac cells were stained and subjected to flow cytometric analyses.

### Flow cytometry.

Isolated cells were resuspended in 2% FBS/PBS solution and blocked with incubation in Fc Block (BD Biosciences) on ice. Immune cell subpopulations were identified by immunostaining for 20 minutes at room temperature using antibodies (Supplemental Table 2) against CD45 (pan-leukocyte marker), CD11b (CD45^+^CD11b^+^, myeloid cells), Ly6G (CD45^+^CD11b^+^Ly6G^+^, neutrophils), F4/80 (CD45^+^CD11b^+^Ly6G^–^F4/80^+^, macrophages) and CD11c (CD45^+^CD11b^+^Ly6G^–^F4/80^–^CD11c^+^, dendritic cells). The 7-aminoactinomycin D high fluorescence dye (7AAD) was added prior to cell analyses to determine cell viability and exclude dead cells. Flow cytometric analyses were performed using a BD FACSAriaIII flow cytometer (BD Biosciences) and FlowJo software.

### Adoptive cell transfer.

Monocytes were isolated from the spleen of hCD68-EGFP^+^ adult mice. Following Schedule 1 killing, the spleen was removed by dissection and the tissue disrupted with the blunt end of a sterile syringe. The disrupted spleen tissue was washed through a 70-μm cell strainer into red cell lysis buffer and incubated at room temperature for 10 minutes. Cells were then spun down, and red cell lysis buffer was removed by aspiration. Monocytes were then purified from the cell pellet using an EasySep Mouse Monocyte Enrichment Kit (STEMCELL Technologies) according to the manufacturer’s instructions. Prior to surgery, i.e., permanent ligation of the LAD, cells were counted and resuspended in sterile PBS. Each recipient animal received 100,000 cells by intramyocardial injection (using a 30G insulin syringe) at the time of MI surgery.

### TUNEL staining.

Apoptotic cells were detected by TUNEL staining in cryosections of infarcted hearts collected on day 7 after MI using the In-Situ Cell Death Detection Kit (Roche) and fluorescence detection, based on the manufacturer’s protocol.

### RNA isolation and gene expression profile by qRT-PCR.

Total RNA was isolated from post-MI heart samples using Trizol reagent (Invitrogen), according to the manufacturer’s instructions. Total RNA was reverse transcribed using oligo-dT primers and Superscript III RT (Invitrogen). qRT-PCR analysis was performed on a ViiA 7 Real-Time PCR System (Thermo Fisher Scientific) using Fast SYBR Green Master Mix (Thermo Fisher Scientific). Data were normalized to *18s*, *Hprt*, and *Gapdh* housekeeping gene expression. Fold change in gene expression was determined by the 2^−ΔΔCT^ method (42). Primer sequences are listed in Supplemental Table 3. Statistical differences were detected using an unpaired, 2-tailed Student’s *t* test.

### Cardiac cine MRI.

Cardiac cine MRI was performed after LAD ligation as described previously (43), with the operator blinded to the experimental group genotype. Briefly, mice were anesthetized with 2% isoflurane in O_2_ and positioned supine in a purpose-built cradle. ECG electrodes were inserted into the forepaws, and a respiration loop was taped across the chest. The cradle was lowered into a vertical-bore, 11.7 T MR System (Magnex Scientific) with a 40-mm birdcage coil (RAPID Biomedical) and a Bruker console running Paravision 2.1.1 (Bruker Medical). A stack of contiguous 1-mm-thick true short-axis ECG-gated cine FLASH images were acquired to cover the entire LV (TE/TR, 1.43/4.6 ms; 17.5° pulse; field of view, 25.6 × 25.6 mm; matrix size, 128 × 128 zero filled to 256 × 256, giving a voxel size of 100 × 100 × 1000 μm; 20–30 frames per cardiac cycle). Long-axis 2-chamber and 4-chamber images were also acquired. Blinded-image analysis was performed using ImageJ (NIH). LV volumes, SV, and EF were calculated as described previously (31).

### Histology.

Upon completion of MRI scanning on day 21 after MI, hearts were harvested and processed for paraffin embedding, cut into Superfrost slides, and deparaffinized using standard methods. For Masson’s trichrome staining (Abcam), sections were stained according to the manufacturer’s instructions. For Picrosirius red staining, sections were stained using the Picro Sirius Red Stain Kit (Abcam) for 60 minutes according to the manufacturer’s protocol and imaged on a Nikon TE2000 microscope under transmission and polarized light. Quantification of fibrotic scarring based on (Picrosirius) yellow-orange birefringence signal was performed using Fiji software.

### Statistics.

Animal numbers and sample sizes reflected the minimal number needed for statistical significance based on prior experience and power analysis calculations to reflect at least a 15% difference in outcome. For MI studies, hearts showing no morphological signs of infarct (e.g., tissue blanching around the ligating suture) at the stage of sample collection were excluded from further analysis. No randomization criteria were used. Statistics were calculated using GraphPad Prism 7 software. The statistical significance between 2 groups was determined using an unpaired 2-tailed Student’s *t* test; these included an *F* test to confirm the 2 groups had equal variances, and the data were reported as mean ± SEM. Among 3 or more groups (e.g., data shown in Figure 6I), 1-way ANOVA followed by Tukey’s multiple comparisons test was used for comparisons. Statistical significance of data generated by the longitudinal cardiac cine MRI approach was determined using 2-way ANOVA with repeated measures. *P* ≤ 0.05 was considered statistically significant.

### Study approval.

All animal experiments were carried out according to United Kingdom Home Office project License PPL30/2987 compliant with the United Kingdom Animals (Scientific Procedures) Act 1986 and approved by the University of Oxford Central Committee on Animal Care and Ethical Review (ACER).

## Author contributions

JMV, SN, and CVdC carried out all experiments (except MI surgeries and flow cytometry), analyzed the data, and contributed figures for the manuscript. TJC, DNB, and JMV performed the flow cytometry studies and analyzed the data generated. MGR performed all MI surgeries. DNB performed histology. CAC performed blinded MRI scanning, and CAC, MGR, and JMV analyzed the MRI data blinded to the genotype of experimental specimen. LAJ and DGJ provided the *Lyve1^–/–^* mice, advice and technical assistance with MLN dissection, and helped edit the manuscript. DRG provided the *hCD68-EGFP* transgenic mice. JMV and PRR established the hypotheses, supervised the studies, and cowrote the manuscript.

## Supplementary Material

Supplemental data

Supplemental Video 1

Supplemental Video 2

## Version 1 07/09/2018

Electronic publication

## Version 2 08/01/2018

Print issue publication
