# Supplementary material for: The cardiac lymphatic system stimulates resolution of inflammation following myocardial infarction
Source: J Clin Invest. 2018 Jul 9;128(8):3402–12. doi: 10.1172/JCI97192 (PMC6063482; doi:10.1172/JCI97192)
Supplement: Supplemental data [file jci-128-97192-s139.pdf]

Supplemental Information

CONTENTS

1. Supplemental Figures 1-9
2. Supplemental Tables 1-3
3. Supplemental Movies 1-2

Vieira et al_Supplemental Figure 1

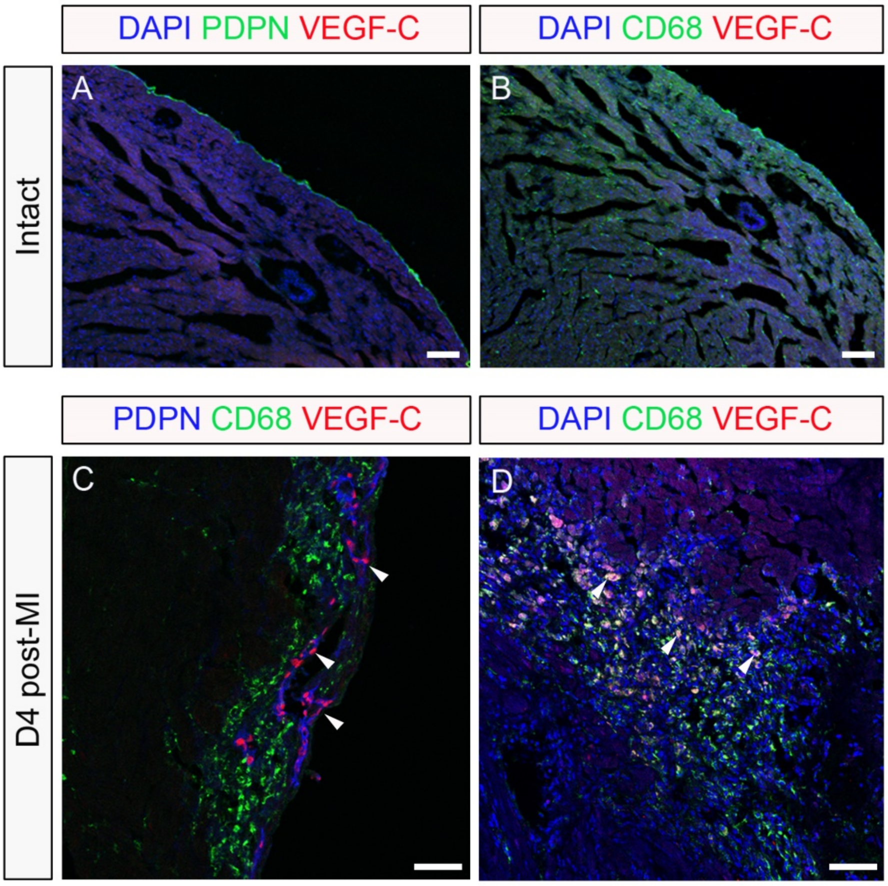

Supplemental Figure 1

Macrophages and epicardial cells are a source of VEGF-C in the injured heart. (**A**) Podoplanin (PDPN; green) and VEGF-C (red) immunostaining of tissue sections derived from intact hearts documenting lack of expression of VEGF-C in the quiescent adult epicardium. (**B**) CD68 (green) and VEGF-C (red) immunostaining of tissue sections derived from intact hearts documenting lack of expression of VEGF-C in $CD68^{+}$ tissue resident macrophages. (**C**) PDPN (blue) and VEGF-C (red) immunostaining of tissue sections derived from day 4 post-MI hearts highlighting expression of VEGF-C in the PDPN-expressing epicardium (white arrowheads). (**D**) CD68 (green) and VEGF-C (red) immunostaining of tissue sections derived from day 4 post-MI hearts showing expression of VEGF-C by $CD68^{+}$ tissue resident macrophages (white arrowheads). DAPI (blue; A, B and D) labels cell nuclei. All scale bars 100 μm.

Vieira et al_Supplemental Figure 2

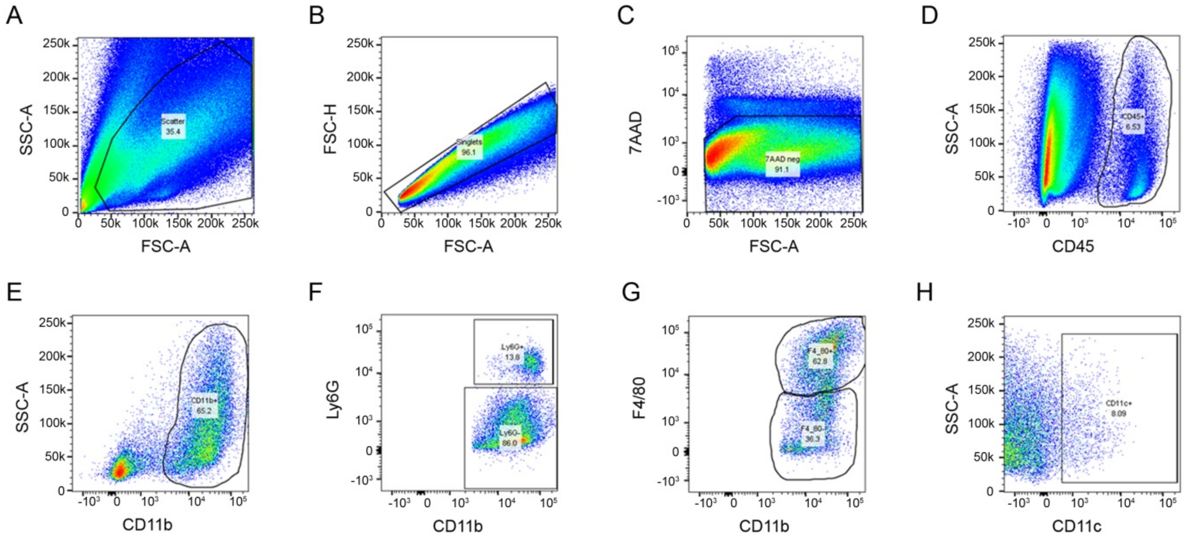

Supplemental Figure 2

Gating strategy for flow cytometry experiments. (**A-H**) Flow cytometry dot plots documenting the gating approach used to measure immune cell populations within the post-MI heart. The 7-aminoactinomycin D high fluorescence dye (7AAD) was used to assess cell viability. Antibodies against CD45 (pan-leukocyte marker), CD11b ($CD45^+/CD11b^+$ = myeloid cells), Ly6G ($CD45^+/CD11b^+/Ly6G^+$ = neutrophils), F4/80 ($CD45^+/CD11b^+/Ly6G^-/F4/80^+$ = macrophages) and CD11c ($CD45^+/CD11b^+/Ly6G^-/F4/80^-/CD11c^+$ = dendritic cell) were used to label the different leukocyte sub-types. Cell populations were expressed as percentages (%) of scatter/singlets from digested post-MI cardiac tissue. This gating approach enabled comparison of immune cell populations between experimental groups. SSC, side scatter; FSC, forward scatter.

Vieira et al_Supplemental Figure 3

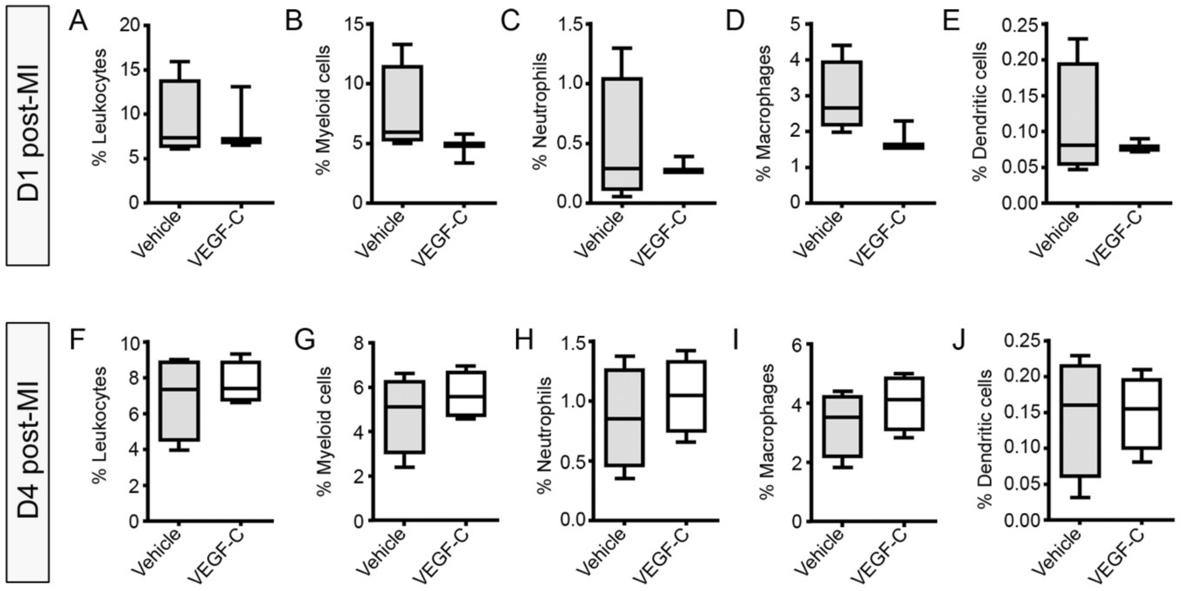

Supplemental Figure 3

Short-course treatment of VEGF-C(C156S) does not modulate the cardiac immune cell content post-injury. (**A**-**J**) Characterization of the immune cell content in vehicle- and recombinant VEGF-C(C156S)-treated hearts at days 1 (**A**-**E**) and 4 (**F**-**J**) post-MI by flow cytometry using antibodies against CD45 (pan-leukocyte marker), CD11b ($CD45^+/CD11b^+$ = myeloid cells), Ly6G ($CD45^+/CD11b^+/Ly6G^+$ = neutrophils), F4/80 ($CD45^+/CD11b^+/Ly6G^-/F4/80^+$ = macrophages) and CD11c ($CD45^+/CD11b^+/Ly6G^-/F4/80^-/CD11c^+$ = dendritic cell). For the D1 post-MI experimental group, a single intraperitoneal (i.p.) injection of vehicle (PBS) or recombinant VEGF-C(C156S) was given at recovery from surgery (day 0) and hearts collected at day 1. For the D4 post-MI experimental group, animals received i.p. injections of vehicle (PBS) or recombinant VEGF-C(C156S) at days 0, 2 and 3 post-MI, with hearts being collected at day 4 post-MI. No significant changes were observed in the % of live immune cells when comparing vehicle- with VEGF-C-treated animals, as determined using an unpaired, two-tailed Student's *t*-test. Data presented as mean ± s.e.m. D1 post-MI: vehicle, n =4 hearts; VEGF-C, n =3 hearts. D4 post-MI: vehicle, n =4 hearts; VEGF-C, n =4 hearts.

Vieira et al_Supplemental Figure 4

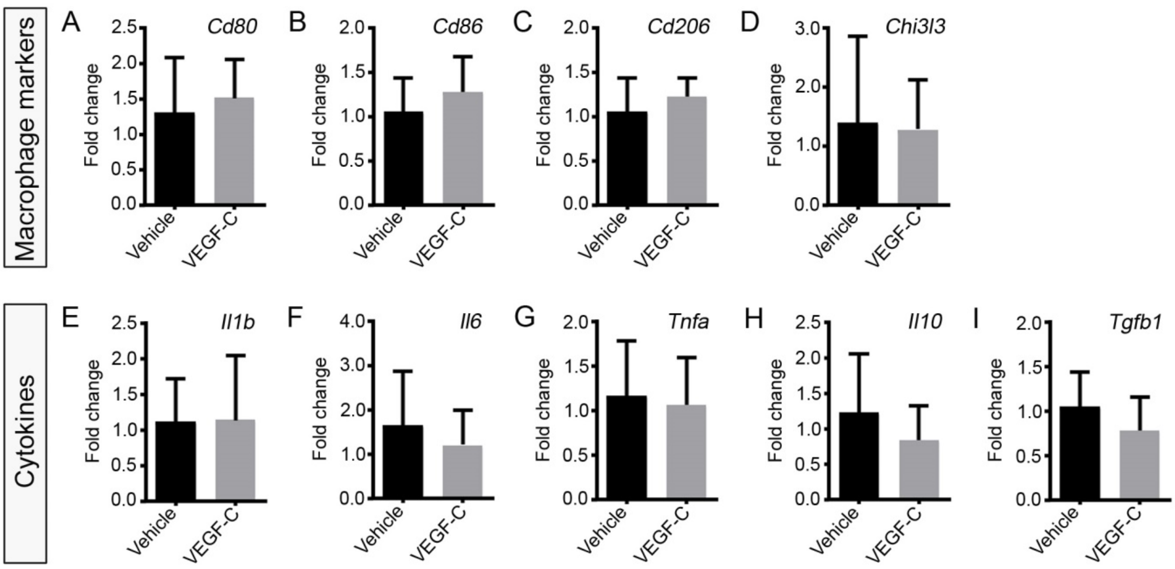

Supplemental Figure 4

Profiling of M1/M2-subtype macrophage subtype markers and inflammatory/reparative cytokines within the infarcted heart. (**A-I**) Marker expression analysis by qRT-PCR using RNA isolated from infarcted cardiac tissue collected at day 7 post-MI from animals that had received intraperitoneal (i.p.) injections of vehicle (PBS) or recombinant VEGF-C(C156S) at days 0, 2, 4 and 6 post-MI. *Cd80* (**A**) and *Cd86* (**B**) are markers of the pro-inflammation M1-subtype macrophages; *Cd206* (**C**) and *Chi3l3* (**D**) are markers of the pro-repair M2-subtype macrophages; *Il1b* (**e**), *Il6* (**F**) and *Tnfa* (**G**) are pro-inflammatory cytokines, *Il10* (**H**) is an anti-inflammatory cytokine and *Tgfb1* (**I**) is a pro-fibrotic cytokine. No significant changes were observed in marker expression between experimental groups as determined using an unpaired, two-tailed Student's *t*-test. Data presented as mean ± s.e.m.; vehicle, *n* =4 hearts; VEGF-C, *n* =5 hearts.

Vieira et al_Supplemental Figure 5

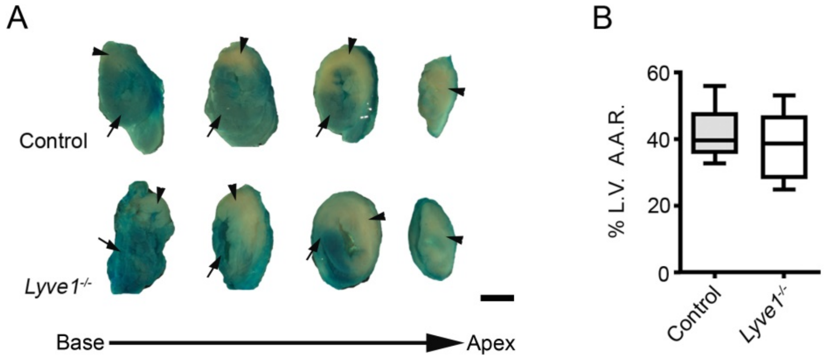

Supplemental Figure 5

Left ventricular area at risk (AAR) following MI is comparable between wildtype and *Lyve1*$^{-/-}$ mice. (A) AAR assessment in transverse sections of control and *Lyve1*$^{-/-}$ hearts following coronary artery ligation. For visualization of the ischemic area, Evans Blue dye is injected into circulation and distributes to the perfused regions (black arrows), but not to the non-perfused (black arrowheads) of the myocardium. **(B)** Quantification of the left ventricular (LV) AAR as % of non-perfused area/ventricule area x 100. Data presented as mean ± s.e.m.; control, n = 5 hearts; *Lyve1*$^{-/-}$, n =5 hearts. No significant differences were found between the two experimental groups using an unpaired, two-tailed Student's t-test. Scale bar 1mm.

Vieira et al_Supplemental Figure 6

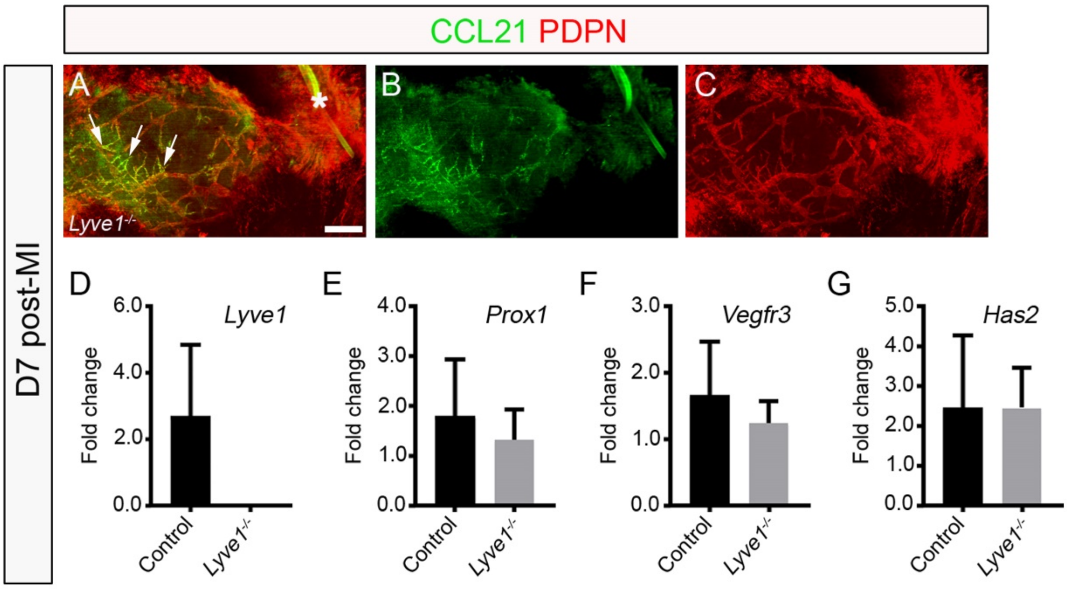

Supplemental Figure 6

***Lyve1$^{-/-}$* hearts exhibit a normal lymphangiogenic response to MI injury.** (**A**-**C**) Whole-mount CCL21 (green) and PDPN (red) immunostaining in *Lyve1$^{-/-}$* adult hearts at day 7 post-MI. White arrows highlight expression of the immune cell chemo-attractant cue CCL21 by PDPN-expressing lymphatic capillaries post-injury. White asterisks indicate the ligating suture. (**D**-**F**) Lymphatic marker expression analysis by qRT-PCR using RNA isolated from infarcted cardiac tissue collected at day 7 post-MI from control and *Lyve1$^{-/-}$* animals. (**G**) Analysis of the hyaluronan synthase 2 (*Has2*) expression by qRT-PCR using RNA isolated from infarcted cardiac tissue collected at day 7 post-MI from control and *Lyve1$^{-/-}$* animals. HAS2 is the main enzyme catalyzing production of hyaluronic acid, the ligand of lymphatic LYVE-1. No significant changes were observed in marker expression between experimental groups as determined using an unpaired, two-tailed Student's *t*-test, with the exception of *Lyve1* that is not expressed in mutants. Data presented as mean ± s.e.m.; control, n =4 hearts; *Lyve1$^{-/-}$*, n =6 hearts. Scale bar 20 µm.

Vieira et al_Supplemental Figure 7

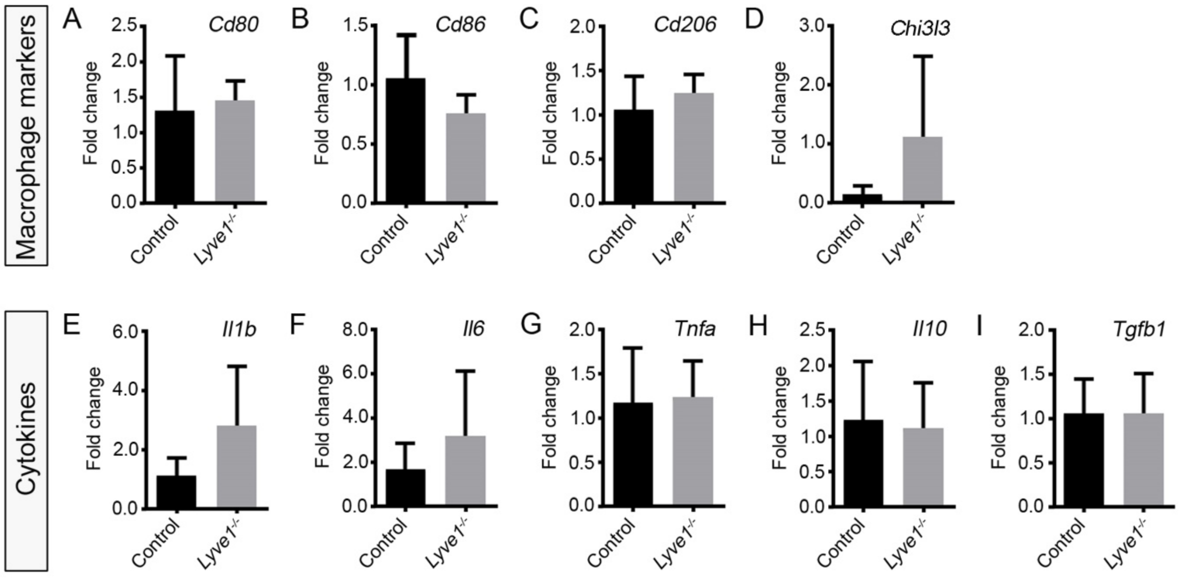

Supplemental Figure 7

Profiling of M1/M2-subtype macrophage subtype markers and inflammatory/reparative cytokines within the infarcted heart of *Lyve1$^{-/-}$* animals. (**A-I**) Marker expression analysis by qRT-PCR using RNA isolated from infarcted cardiac tissue collected at day 7 post-MI from control and *Lyve1$^{-/-}$* animals. *Cd80* (**A**) and *Cd86* (**B**) are markers of the pro-inflammation M1-subtype macrophages; *Cd206* (**C**) and *Chi3l3* (**D**) are markers of the pro-repair M2-subtype macrophages; *Il1b* (**E**), *Il6* (**F**) and *Tnfa* (**G**) are pro-inflammatory cytokines, *Il10* (**H**) is an anti-inflammatory cytokine and *Tgfb1* (**I**) is a pro-fibrotic cytokine. No significant changes were observed in marker expression between experimental groups as determined using an unpaired, two-tailed Student's *t*-test. Data presented as mean ± s.e.m.; control, *n* =4 hearts; *Lyve1$^{-/-}$*, *n* =6 hearts.

Vieira et al_Suplemental Figure 8

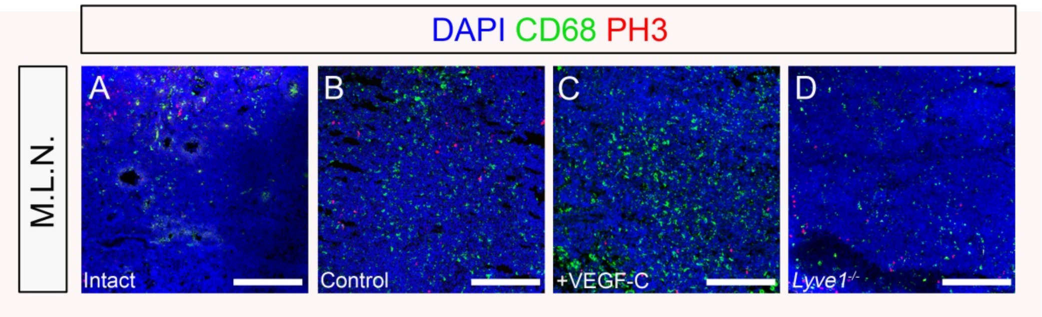

Supplemental Figure 8

Immune cells in mediastinal lymph nodes do not undergo proliferation post-myocardial infarction. (**A**-**D**) CD68 (green) and PH3 (red) immunostaining of tissue sections derived from intact (no MI; **A**); control (**B**), recombinant VEGF-C(C156S)-treated (**C**) and *Lyve1*$^{-/-}$ (**D**) mediastinal lymph nodes (MLNs) collected 7 days post-MI. Note the relative excess of CD68^{+} macrophages in VEGF-C-treated MLNs, compared to *Lyve1*$^{-/-}$ and controls (compare **C** with **D** and **B**). No CD68^{+}/PH3^{+} phagocytic cells were found, suggesting that augmented CD68^{+} cell population in VEGF-C-treated MLNs is not due to a proliferative response of tissue-resident macrophages. DAPI (blue) labels cell nuclei. All scale bars 100 µm.

Vieira et al_Suplemental Figure 9

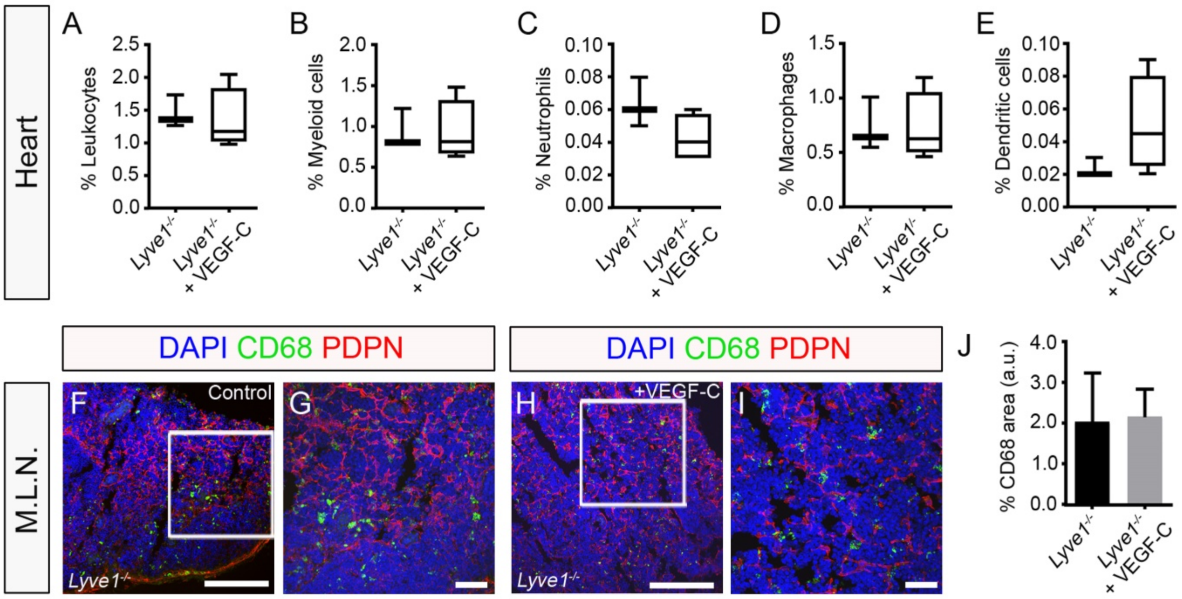

Supplemental Figure 9

VEGF-C treatment does not improve immune cell clearance in *Lyve1*$^{-/-}$ after myocardial infarction. (**A-E**) Characterization of the immune cell content in hearts derived from *Lyve1*$^{-/-}$ and *Lyve1*$^{-/-}$ treated with recombinant VEGF-C(C156S) at day 7 post-MI by flow cytometry using antibodies against CD45 (pan-leukocyte marker), CD11b (CD45^{+}/CD11b^{+}= myeloid cells), Ly6G (CD45^{+}/CD11b^{+}/Ly6G^{+}= neutrophils), F4/80 (CD45^{+}/CD11b^{+}/Ly6G^{-}/F4/80^{+}= macrophages) and CD11c (CD45^{+}/CD11b^{+}/Ly6G^{-}/F4/80^{-}/CD11c^{+}= dendritic cell). Animals received intraperitoneal (i.p.) injections of vehicle (PBS; *Lyve1*$^{-/-}$) or recombinant VEGF-C(C156S) (*Lyve1*$^{-/-}$ +VEGF-C) at days 0, 2, 4 and 6 post-MI. Data presented as mean ± s.e.m.; control, n =4 hearts; *Lyve1*$^{-/-}$, n =4 hearts. No significant changes were found between the two experimental groups using an unpaired, two-tailed Student's t-test. (**F-I**) CD68 (green) and PDPN (red) immunostaining of tissue sections derived from mediastinal lymph nodes (MLNs) of *Lyve1*$^{-/-}$ (control vehicle-treated; **F** and **G**) and *Lyve1*$^{-/-}$ treated with recombinant VEGF-C(C156S) (+VEGF-C; **H** and **I**), collected 7 days post-MI. (**G**) Magnified view of box shown in (**F**); (**I**) magnified view of box shown in (**H**). DAPI (blue) labels cell nuclei. (**J**) Quantification of macrophage proportion as % CD68^{+} total staining area/total DAPI-labelled tissue area x 100. Data presented as mean ± s.e.m.; a.u., arbitrary units; *Lyve1*$^{-/-}$, n =4 MLNs; *Lyve1*$^{-/-}$ +VEGF-C, n =4 MLNs. Note that one MLN was analyzed per mouse. No significant differences were found between the two experimental groups using an unpaired, two-tailed Student's t-test. Scale bars **F** and **H** 100 µm; **G** and **I** 50 µm.

Supplemental Table 1. Functional left ventricle parameters from the cardiac cine-MRI study using control and *Lyve1*$^{-/-}$ animals following MI

	**7 days post-MI**		**21 days post-MI**	
	Control (n =10)	***Lyve1*$^{-/-}$ *(n = 10)***	***Control (n =10)***	***Lyve1*$^{-/-}$ *(n = 10)***
End diastolic volume (μL)	62.47 ± 8.52	62.18 ± 6.23	74.26 ± 9.26	72.69 ± 8.15
End systolic volume (μL)	36.47 ± 8.26	**34.34 ± 6.17***	43.33 ± 9.00	**47.18 ± 7.92***
Stroke volume (μL)	**26.00 ± 1.25***	27.85 ± 1.43	**30.93 ± 1.15***	**25.52 ± 1.84***
Ejection fraction (%)	46.91 ± 5.15	**47.98 ± 4.48****	**45.86 ± 4.26***	**38.45 ± 4.69***
Cardiac output (mL/min)	12.30 ± 0.60	13.04 ± 0.67	14.73 ± 0.76	12.05 ± 0.85
Ventricular mass (mg)	97.84 ± 8.96	84.37 ± 4.94	111.6 ± 8.14	96.27 ± 5.34
Absolute infarct size (mm^2)	22.68 ± 6.54	22.42 ± 5.37	16.93 ± 5.77	18.98 ± 5.05
Relative infarct size (%)	**19.21 ± 4.86***	18.41 ± 4.70	**13.35 ± 3.70***	17.54 ± 4.10

Data presented as mean ± standard error of the mean. Asterisks indicate significant differences between experimental groups, as determined by two-way ANOVA with repeated measures (*$P \leq 0.05$; **$P \leq 0.01$).

Supplemental Table 2. List of antibodies and applications

**Immunostaining**		
**Antibody**	**Company/Cat. No.**	**Dilution**
Goat anti-CCL21	R&D Systems (AF457)	1:100
Rabbit anti-LYVE-1	Angiobio (11-034)	1:200
Rabbit anti-VEGF-C	Abcam (ab9546)	1:200
Goat anti-VEGFR-3	R&D Systems (AF743)	1:50
Hamster anti-PDPN	Stratech (10R-P155A)	1:500
Rat anti-CD68	Bio-Rad (MCA1957)	1:500
Rabbit anti-p-Histone H3 (PH3)	Santa Cruz (sc-8656-R)	1:100
**Flow cytometry**		
**Antibody/Fluorophore**	**Company/Cat. No.**	**Dilution**
Rat anti-CD45 (AlexaFluor647)	BioLegend Inc. (103124)	1:200
Rat anti-CD11b (FITC)	BioLegend Inc. (101206)	1:100
Rat anti-Ly6G (Brilliant Violet 421)	BioLegend Inc. (127636)	1:200
Rat anti-F4/80 (PE)	BioLegend Inc. (123110)	1:100
Rat anti-CD11c (APC/Cy7)	BioLegend Inc. (117323)	1:100

Supplemental Table 3. List of primers used in qRT-PCR studies

Gene	Forward (5'-3')	Reverse (5'-3')
18s	CATTCGAACGTCTGCCCTAT	GTTTCTCAGGCTCCCTCTCC
Cd206	CAGGTGTGGGCTCAGGTAGT	TGTGGTGAGCTGAAAGGTGA
Cd80	GGCAAGGCAGCAATACCTTA	CTCTTTGTGCTGCTGATTCG
Cd86	CTTACGGAAGCACCCATGAT	CCCATTGAAATAAGCTTGCG
Chi3l3	AGGAGCAGGAATCATTGACG	TTTCTCCAGTGTAGCCATCCTT
Gapdh	AGGTCGGTGTGAACGGATTTG	TGTAGACCATGTAGTTGAGGTCA
Hprt	TCAGTCAACGGGGGACATAAA	GGGGCTGTACTGCTTAACCAG
Il10	GGTTGCCAAGCCTTATCGGA	ACCTGCTCCACTGCCTTGCT
Il1b	AAAGAATCTATACCTGTCCTGTGTAATGAAA	GGTATTGCTTGGGATCCACACT
Il6	CTTCCATCCAGTTGCCTTCTTG	AATTAAGCCTCCGACTTGTGAAG
Lyve1	GGCTTTGAGACTTGCAGCTATG	GCAGGAGTTAACCCAGGTG
Prox1	GAAGGGCTATCACCCAATCA	TGAACCACTTGATGAGCTGC
Tgfb1	CCGCTGCATATCGTCCTGTG	AGTGGATGGATGGTCCTATTACA
Tnfa	CTGTAGCCCACGTCGTAGC	TTGAGATCCATGCCGTTG
Vegfr3	CCATCGAGAGTCTGGACAGC	CCGGGATGGTGGTCACATAG

Supplemental Movie 1. Representative stack of contiguous 1-mm thick true short-axis cine-FLASH images of a control heart at day 21 post-MI acquired by MRI.

Supplemental Movie 2. Representative stack of contiguous 1-mm thick true short-axis cine-FLASH images of a *Lyve1*$^{-/-}$ heart at day 21 post-MI acquired by MRI.
